# Modulation of viral replication, apoptosis and antiviral response by induction and mutual regulation of EGR and AP-1 family genes during coronavirus infection

**DOI:** 10.1080/22221751.2022.2093133

**Published:** 2022-07-04

**Authors:** Lixia Yuan, To Sing Fung, Jiawen He, Rui Ai Chen, Ding Xiang Liu

**Affiliations:** a Integrative Microbiology Research Centre, South China Agricultural University, Guangzhou, People’s Republic of China; b Zhaoqing Branch Center of Guangdong Laboratory for Lingnan Modern Agricultural Science and Technology, Zhaoqing, People’s Republic of China

**Keywords:** Coronavirus, immediate-early genes, EGR1, AP-1, ERK1/2, cross-activation, cytokines, apoptosis

## Abstract

Coronaviruses have evolved a variety of strategies to exploit normal cellular processes and signalling pathways for their efficient reproduction in a generally hostile cellular environment. One immediate-early response gene (IEG) family, the AP-1 gene family, was previously shown to be activated by coronavirus infection. In this study, we report that another IEG family, the EGR family, is also activated in cells infected with four different coronaviruses in three genera, i.e. gammacoronavirus infectious bronchitis virus (IBV), alphacoronaviruses porcine epidemic diarrhoea virus (PEDV) and human coronavirus-229E (HCoV-229E), and betacoronavirus HCoV-OC43. Knockdown of EGR1 reduced the expression of cJUN and cFOS, and knockdown of cJUN and/or cFOS reduced the expression of EGR1, demonstrating that these two IEG families may be cross-activated and mutual regulated. Furthermore, ERK1/2 was identified as an upstream kinase, and JNK and p38 as inhibitors of EGR1 activation in coronavirus-infected cells. However, upregulation of EGR family genes, in particular EGR1, appears to play a differential role in regulating viral replication, apoptosis and antiviral response. EGR1 was shown to play a limited role in regulation of coronavirus replication, and an anti-apoptotic role in cells infected with IBV or PEDV, but not in cells infected with HCoV-229E. Upregulation of EGR1 may also play a differential role in the regulation of antiviral response against different coronaviruses. This study reveals a novel regulatory network shared by different coronaviruses in the immediate-early response of host cells to infection.

## Introduction

Coronaviruses are a family of single-stranded, positive sense RNA viruses that cause severe diseases in humans and animals. The current pandemic of coronavirus disease-19 (COVID-19), caused by severe acute respiratory syndrome coronavirus-2 (SARS-CoV-2), has posed an unprecedented threat to human life and social development [1]. The presence of multiple human and animal coronavirus species, in combination with their large genome and a complex replication cycle, makes it difficult to understand the interactions between these viruses and their host cells [2,3]. On the other hand, revelation of a fuller picture of such interactions, especially some general cellular pathways and processes shared by different coronaviruses would be essential for a deep understanding of the replication mechanisms and pathogenesis of these viruses of medical and veterinary importance, and meanwhile of help to the identification of potentially new therapeutic targets.

Immediate-early genes (IEGs) are a group of genes that are first expressed in response to various external stimuli, such as serum, growth factors, cytokines, tumour promoters, ultraviolet radiation, hormones and stress [4–6]. A variety of IEGs have been identified, including mainly AP-1 family genes (FOS and JUN), EGR, MYC and others [7]. Most IEGs encode transcription factors that regulate genes involved in various cell functions in normal cell growth and differentiation, intracellular information transmission and energy metabolism [8]. EGR1, also known as NGFi-A, Krox24, ZIF268 and TIS8, is one of the EGR family genes and belongs to IEGs [9]. EGR1 expression can be induced by a variety of stimuli, including growth factors, cytokines, mitogen, apoptosis, stress and tissue damage [10]. In response to external stimuli, EGR1 transmits signals downstream to activate specific target genes with different functions, such as regulation of cyclins, cytokines, transcription factors and growth factors [11–13]. EGR1 activation can be mediated by mitogen-activated protein kinase (MAPK) cascade and protein kinase A (PKA) as upstream signalling pathways [14]. It has been shown that MAPK pathway plays an important role in inducing EGR1 expression in response to endoplasmic reticulum (ER) stress [15]. In addition, EGR1 plays a regulatory role in inflammation and immune response, and is a key factor in the production of proinflammatory cytokines, such as interleukins (IL) and tumour necrosis factor (TNF), and chemokines [11,16,17]. EGR family genes include EGR1, EGR2, EGR3 and EGR4. Both EGR family genes and AP-1 family genes are immediate early response factors. The involvement of AP-1 family genes in coronavirus biology have been studied in our previous reports [18–20], however, the role of EGR family genes in coronavirus infection, especially the detailed mechanisms of EGR1-activation in coronavirus-induced apoptosis and the relationship between AP-1 and EGR family genes in coronavirus-infected cells, remains unclear.

Our previous studies have shown that coronavirus infection induced the expression of AP-1 family genes cFOS and cJUN [18–20]. In this study, we report the upregulation of EGR1 in cells infected with coronavirus infectious bronchitis virus (IBV), as revealed by transcriptomic analysis and subsequent verification by RT-qPCR and Western blotting. The EGR family genes are confirmed to be significantly upregulated in cells infected with IBV, porcine epidemic diarrhoea virus (PEDV), human coronavirus-229E (HCoV-229E) and HCoV-OC43, respectively, under the positive regulation of ERK1/2 and negative regulation of JNK and p38. Furthermore, the coronavirus infection-induced upregulation of cFOS/cJUN and ERG1 is mutually regulated and plays a functional role in regulation of viral replication, apoptosis and antiviral response. This study reveals a novel cellular regulatory network in the early response to coronavirus infection and provides a new direction for further elucidation of the relationship between coronaviruses and their hosts.

## Materials and methods

### Virus and cells

The egg-adapted Beaudette strain of IBV (ATCC VR-22) was obtained from the American Type Culture Collection (ATCC) and adapted to Vero cells as previously described [21,22]. PEDV virulent strain DR13 (GenBank accession no. JQ023162) was isolated from a suckling pig in 1999, and adapted to growth in Vero cells [23,24]. HCoV-229E (accession No. KU291448.1) [25] and HCoV-OC43 (accession No. KU131570.1) [26] were obtained from ATCC. Cell culture, virus stock preparation, UV-inactivation of viruses and IBV infection of chicken embryos were carried out as previously described [19].

### Antibodies, chemicals and reagents

Antibodies against EGR1 (#4154), cJUN (#9165), cFOS (#2250), ERK1/2 (#9194), JNK (#9252), p38 (#9212), PARP (#9532), β-actin (#4967) were purchased from Cell Signaling Technology. Goat anti-rabbit IgG H&L (Alexa Fluor® 488) (ab150077) was purchased from Abcam. Antisera against IBV N protein were prepared in rabbits immunized with bacterially expressed fusion proteins as previously described [27,28]. MEK inhibitor U0126, p38 inhibitor SB203580 and JNK inhibitor SP600125 were purchased from Selleckchem.

### Cell viability assay

Cell viability assay was performed according to the manufacturer’s instructions from TransDetect CCK as previously described [19].

### Transcriptomic analysis

Transcriptomic analysis was carried out by the Biomarker Technologies Co, LTD, Beijing, China as previously described [19].

### RNA interference

The sequences of the siRNA for EGFP, cJUN, JNK, p38, ERK1/2 and cFOS were previously described [19], and sequences of the siRNA strands for human EGR1, cJUN [20], JNK [20] and p38 [29] are listed in table S1. Transfection of siRNA was performed using the TransIntro EL transfection reagent (TransGen Biotech) as previously described [18,19].

### Plasmid construction and transfection

The complementary DNA (cDNA) of human EGR1 (NM_001964) was amplified from total RNA of IBV-infected H1299 cells by reverse transcription-polymerase chain reaction (RT–PCR), using primer pair as listed in table S2. The PCR product was inserted to pXJ40-Flag by homologous recombination. The plasmid was confirmed by nucleotide sequencing and named pXJ40-Flag-EGR1. Plasmid DNA was transfected into H1299 cells using TransIntro EL transfection regent (Transgen biotech) as previously described [18].

### RNA extraction and RT-qPCR analysis

RNA extraction and RT-qPCR analysis were performed as previously described [18,19]. The qPCR primers used in this study are listed in table S2.

### SDS-PAGE and Western blot analysis

SDS-PAGE and Western blot analysis were performed as previously described [18,19]. All experiments were repeated at least three times with similar results, and one of the representative results was shown.

Cleavage of Poly ADP-Ribose polymerase (PARP), a DNA repair enzyme and the substrate of activated casparases, is a hallmark of apoptosis and caspase activation. Percentage of PARP cleavage [PARP Clv. (%)] was calculated as the intensity of cleaved PARP (Cl) divided by the total intensity of the full-length PARP (FL) + Cl.

### Statistical analysis

The one-way ANOVA method was used to analyse the significant difference between the indicated sample and the respective control sample. Significance levels were presented by the *p*-value in all figures (ns, non-significant; *p < 0.05; **p < 0.01; ***p < 0.001).

## Result

### Upregulation of EGR family by infection of cells and/or chicken embryos with IBV, PEDV, HCoV-229E and HCoV-OC43

Transcriptomic analysis of IBV-infected H1299 cells showed that IBV infection induced the expression of EGR family genes, EGR1, EGR2, EGR3 and EGR4. As summarized in Table S3, EGR1 was induced by a 117-fold, EGR2 was induced by a 68-fold, EGR3 was induced by a 122-fold, and EGR4 was induced by a 261-fold (Table S3).

**Figure 1.**
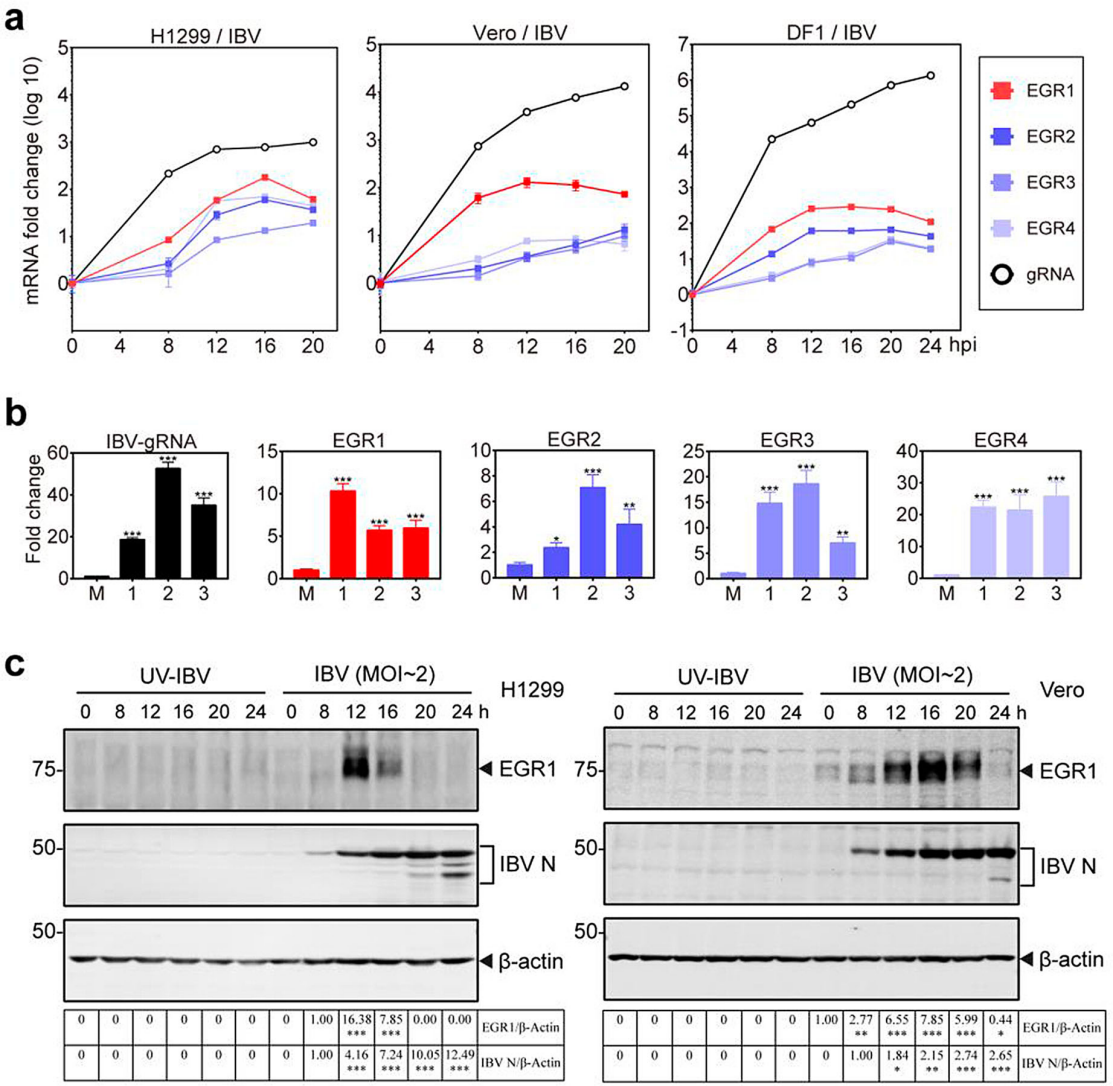
Upregulation of EGR family genes in IBV-infected cells and chicken embryos. (a) Upregulation of EGR family genes at the mRNA level in IBV-infected H1299, Vero and DF1 cells. Cells were infected with IBV (MOI∼2), and harvested at indicated time points. The levels of virus genomic RNA (gRNA) and the mRNA levels of EGR family genes (EGR1/EGR2/EGR3/EGR4) were determined by qPCR. (b) Upregulation of EGR family genes at the mRNA level in IBV-infected chicken embryos. Ten-day-old SPF chicken embryos were inoculated with 200 µL of IBV (500 PFU). At 60 hpi, chicken embryo viscera were collected. The EGR family genes (EGR1/EGR2/EGR3/EGR4) at the mRNA level as well as IBV gRNA were determined by qPCR. Shown are the results of three repeated experiments, as indicated. (c) Upregulation of EGR1 at the protein level in IBV-infected H1299 and Vero cells. H1299 and Vero cells were infected with IBV (MOI∼2), or UV-IBV. Cell lysates were harvested at the indicated time points and subjected to Western blot analysis using indicated antibodies. Sizes of protein ladders in kDa were indicated on the left.

To verify the transcriptomic data and to investigate if upregulation of EGR family genes was a common mechanism during IBV infection of different culture cells and chicken embryos, detailed time course experiments were firstly conducted in H1299, Vero and DF1 cells infected with IBV, respectively. The results showed that all the four EGR family genes were upregulated at 8 hpi in IBV-infected H1299 cells, and reached the peak at 16 hpi (Figure 1(a)). In IBV-infected Vero cells, EGR1 and EGR4 reached the peak at 12 hpi, while EGR2 and EGR3 reached the peak at 20 hpi (Figure 1(a)). In IBV-infected DF1 cells, EGR1 and EGR2 reached the peak at 12 hpi, while EGR3 and EGR4 reached the peak at 20 hpi (Figure 1(a)). Upregulation of EGR family genes was also observed in IBV-infected chicken embryos (Figure 1(b)). Western blot analysis of the induction kinetics of EGR1 at the protein level was then conducted in IBV-infected H1299 and Vero cells. The expression of EGR1 protein was induced in the infected H1299 and Vero cells at 8 hpi, reaching the peak at 12 and 16 hpi, respectively (Figure 1(c)). These results confirm that IBV infection induces the expression of EGR family genes at the mRNA and protein levels.

To test if the upregulation of EGR family genes was a common mechanism shared by other coronaviruses, similar time course experiments were conducted in H1299 and/or Vero cells infected with PEDV, HCoV-229E and HCoV-OC43, respectively. Upregulation of these four EGR family genes at the protein and mRNA levels was also observed in PEDV, HCoV-229E and HCoV-OC43-infected cells (Figure S1a,b). Taken together, these results demonstrate that induction of the expression of EGR family genes is a general mechanism during coronaviruses infection, suggesting that genes in this family may play important roles in regulating coronavirus replication and virus-host interaction.

### Functional significance of EGR1 induction in regulating coronavirus replication and apoptosis

**Figure 2.**
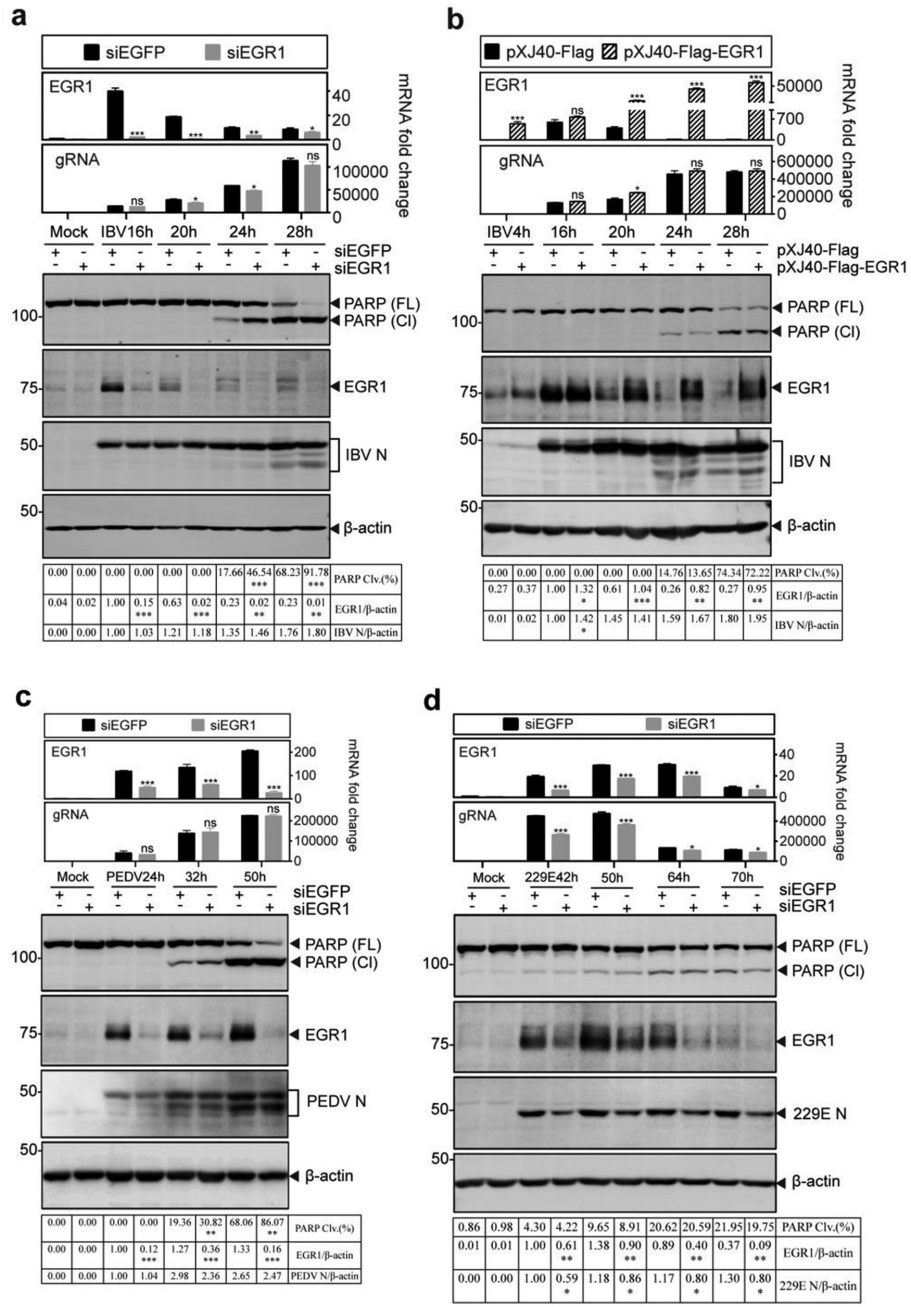
Functional significance of EGR1 upregulation on coronavirus replication and apoptosis (a) Promotion of IBV-induced apoptosis by knockdown of EGR1. H1299 cells were transfected with siEGFP and siEGR1, before infected with IBV (MOI∼2). Cells were harvested at the indicated time points and subjected to RT-qPCR and Western blot analysis, respectively. The mRNA levels of EGR1 and IBV gRNA were determined by qPCR. Western blot analysis was performed using the indicated antibodies. Sizes of protein ladders in kDa were indicated on the left. Percentage of PARP cleavage [PARP Clv. (%)] was calculated as the intensity of cleaved PARP (Cl) divided by the total intensities of the full-length PARP (FL) + Cl. (b) Inhibition of IBV-induced apoptosis by overexpression of EGR1. H1299 cells were transfected with pXJ40-Flag and pXJ40-Flag-EGR1, respectively before being infected with IBV (MOI∼2). Cell lysates were prepared and analysed as (a). The mRNA levels of EGR1 and IBV gRNA were determined by qPCR. Western blot was performed using antibodies against EGR1, IBV N, and PARP. (c) Effects of EGR1-knockdown on PEDV-induced apoptosis. H1299 cells were treated as (a), before infected with PEDV (MOI∼2). Cell lysates were prepared and analysed as (a). (d) Effects of EGR1-knockdown on HCoV-229E-induced apoptosis. H1299 cells were treated, before infected with HCoV-229E (MOI∼2), lysates prepared and analysed as (a).

To investigate if EGR1 is functionally involved in the regulation of coronavirus replication and coronavirus infection-induced apoptosis, knockdown of EGR1 with small interfering RNA (siRNA) in H1299 cells was carried out, and the knockdown cells were then infected with IBV, PEDV and HCoV-229E, respectively. In IBV-infected cells, knockdown of EGR1 does not significantly affect IBV replication, as similar levels of IBV N protein and viral genomic RNA were detected in both knockdown and control cells (Figure 2(a)). Interestingly, significantly higher percentages of PARP cleavage were detected in EGR1-knockdown cells infected with IBV than that in the control cells at 24 and 28 hpi (Figure 2(a)), suggesting that EGR1 may function as a survival factor during IBV infection of H1299 cells.

The effect of EGR1 expression on IBV replication and apoptosis was further tested by overexpression of a Flag-tagged EGR1 construct in H1299 cells before IBV infection. As shown in Figure 2(b), the overexpressed EGR1 protein and higher levels of EGR1 mRNA were detected in the transfected cells. The levels of IBV N protein and viral genomic RNA were slightly increased in EGR1-overexpressing cells (Figure 2(b)), suggesting that overexpression of EGR1 renders minor effects on the replication of IBV. This is probably due to the drastic induction of the endogenous EGR1 (as well as other members of the EGR family proteins) during IBV infection, which might reach its functional threshold in a given cell and offset the impact of the overexpressed EGR1. Consistent with results obtained from the knockdown cells, slightly less PARP cleavage was detected in the infected cells overexpressing EGR1 (Figure 2(b)).

Knockdown of EGR1 slightly reduced the replication of PEDV in H1299 cells, as slightly less N protein and viral genomic RNA were detected in the knockdown cells, compared with the control (Figure 2(c)). Interestingly, significantly more PARP cleavage was observed in the knockdown cells at 32 and 50 hpi (Figure 2(c)). Infection of EGR1-knockdown cells with HCoV-229E showed significantly reduced detection of viral protein and genomic RNA as well as PARP cleavage (Figure 2(d)). These results confirm that EGR1 plays a differential regulatory role in coronavirus replication and, meanwhile, functions as a survival factor in IBV- and PEDV-induced apoptosis.

### Mutual regulation of the coronavirus-infection induced EGR1 and cFOS/cJUN expression

**Figure 3.**
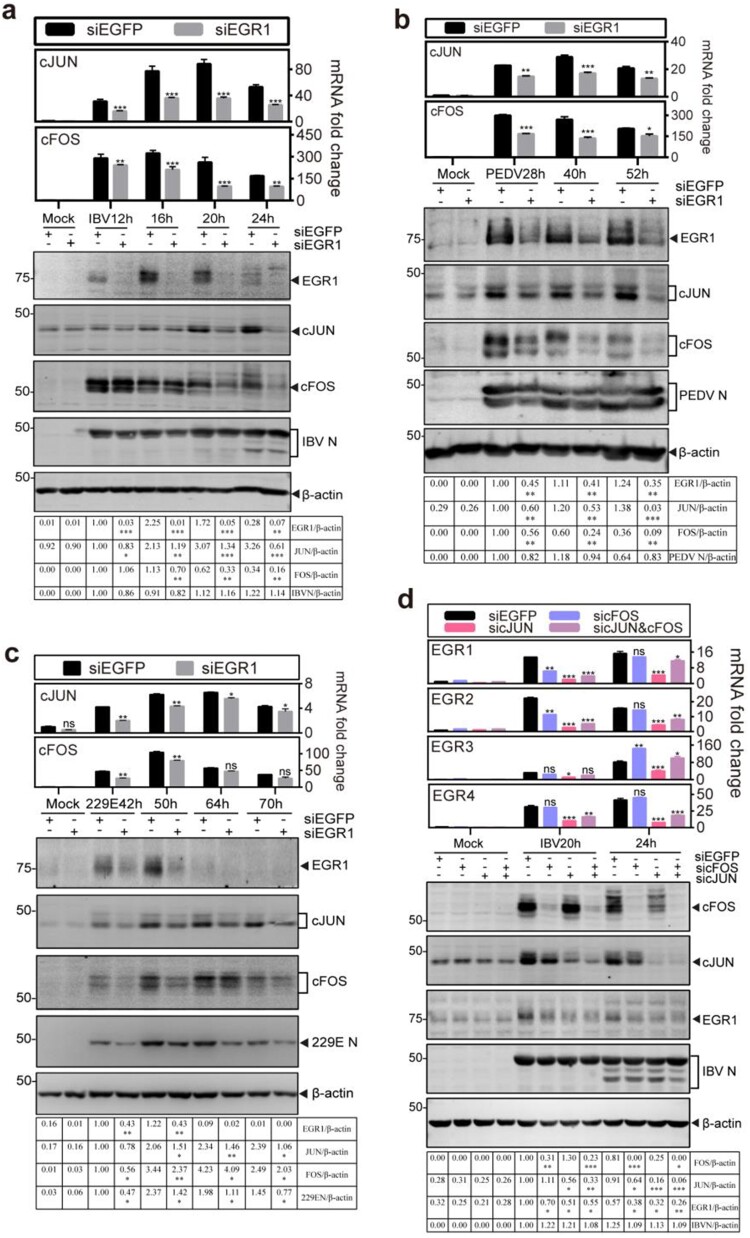
Mutual regulation of the coronavirus infection-induced EGR1 and cFOS/cJUN expression. (a) Down-regulation of cFOS/cJUN expression by knockdown of EGR1 in IBV-infected cells. H1299 cells were transfected with siEGFP and siEGR1, before infected with IBV. Cells were harvested at the indicated time points and subjected to RT-qPCR and Western blot analysis, respectively. The mRNA levels of cJUN and cFOS were determined by qPCR. Western blot analysis was performed using the indicated antibodies. Sizes of protein ladders in kDa were indicated on the left. (b) Down-regulation of cFOS/cJUN expression by knockdown of EGR1 in PEDV-infected cells. H1299 cells were treated before infected with PEDV, lysates prepared and analysed as (a). (c) Down-regulation of cFOS/cJUN expression by knockdown of EGR1 in HCoV-229E-infected cells. H1299 cells were treated before infected with HCoV-229E, lysates prepared and analysed as (a). (d) Down-regulation of EGR1 expression by knockdown of cFOS and/or cJUN in IBV-infected cells. H1299 cells were transfected with siEGFP, sicJUN, sicFOS and sicJUN&sicFOS, before infected with IBV. Cell lysates were prepared and analysed as (a). The mRNA levels of EGR family genes (EGR1/EGR2/EGR3/EGR4) were determined by qPCR. Western blot analysis was performed as (a).

As both AP-1 family and EGR family genes are IEGs, it would be interesting to test if mutual regulation may occur between the two family genes. For this purpose, the effect of EGR1-knockdown on the expression of cFOS and cJUN after IBV infection was firstly studied in time-course experiments. As shown in Figure 3(a), knockdown of EGR1 drastically reduced the induction of cJUN at both mRNA and protein levels throughout the time course. Minor to moderate inhibitory effects on cFOS induction were observed at 16 and 20 hpi, but more drastic inhibition was observed at later time points (Figure 3(a)). Similar effects on the induction of cJUN and cFOS were also observed in EGR1-knockdown cells infected with PEDV (Figure 3(b)) and HCoV-229E (Figure 3(c)), respectively.

The effects of knockdown of cFOS and cJUN on the expression of EGR family genes were then studied in the knockdown cells infected with IBV. As shown in Figure 3(d), knockdown of either cFOS or cJUN significantly reduced the EGR1 protein expression, and even less EGR1 protein was detected in both cFOS- and cJUN-knockdown cells. Consistently, the EGR1 mRNA was significantly decreased in the knockdown cells, especially at 20 and 24 hpi in cJUN-knockdown cells (Figure 3(d)). Varying inhibitory effects on the expression of EGR2, EGR3 and EGR4 genes at the mRNA level were detected in the knockdown cells (Figure 3(d)). These data demonstrate that both AP-1 and EGR family genes are activated by coronavirus infection and the activation of these two family genes in the infected cells is mutually regulated, prompting further study on the upstream kinases that may regulate EGR1 expression.

### ERK1/2 functioning as an upstream kinase in coronavirus infection-induced upregulation of EGR1

**Figure 4.**
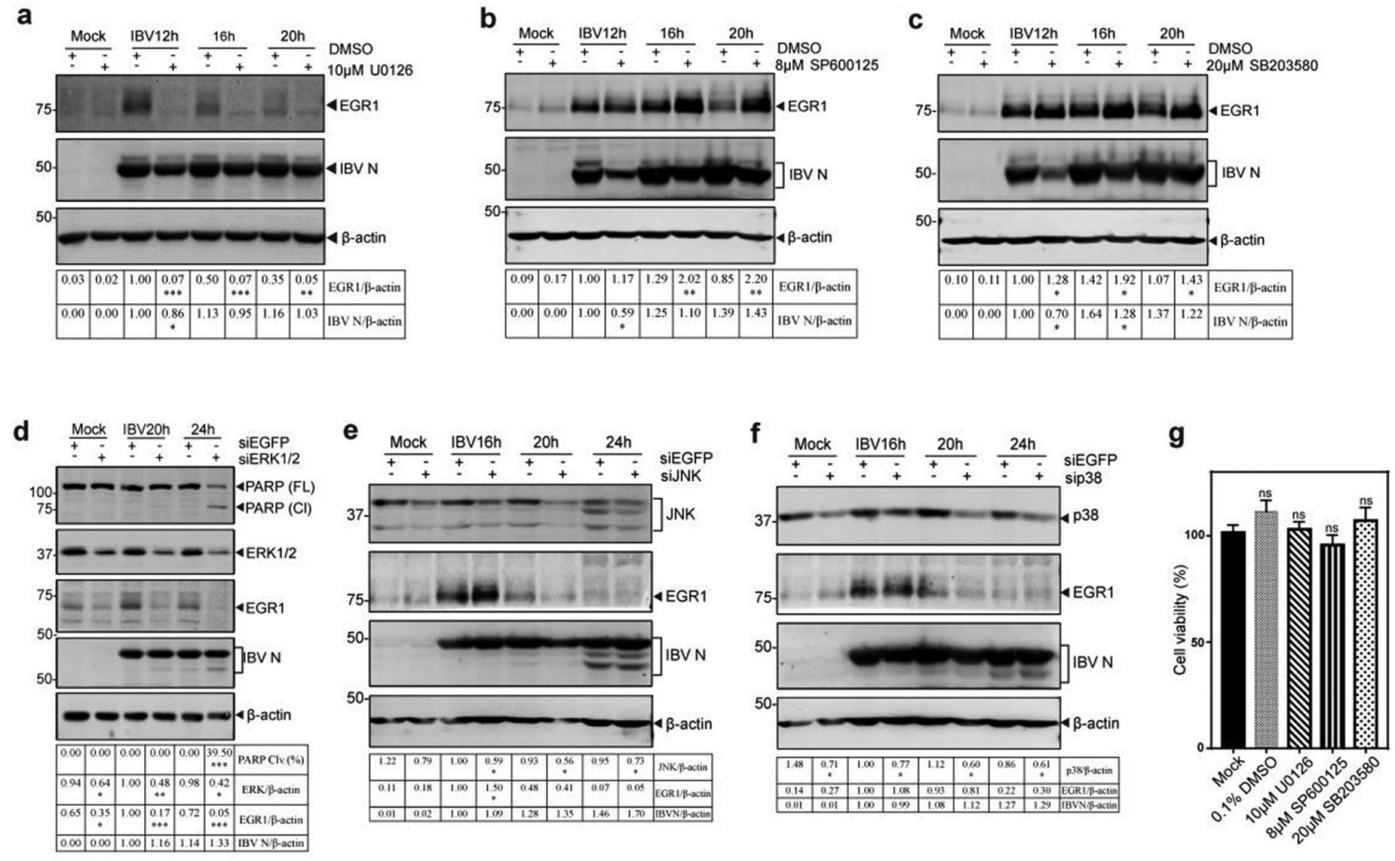
Identification of ERK1/2 as an upstream kinase(s) for EGR1 induction in IBV-infected cells. (a/b/c) Identification of the upstream kinase(s) MAPK for EGR1 induction in IBV-infected cells. H1299 cells were infected with IBV and treated with MAPK/ERK1/2/JNK/p38 inhibitor U0126, SP600125 and SB03580 at the indicated concentrations or with the same volume of DMSO at 2 hpi, respectively, Cells were harvested at the indicated time points. Western blot analysis was performed using the indicated antibodies. Sizes of protein ladders in kDa were indicated on the left. (d/e/f) Effects of ERK1/2-, JNK- or p38-knockdown on the expression of EGR1 in IBV-infected cells. H1299 cells were transfected with siEGFP and siERK1/2/JNK/p38, before infected with IBV. Cells were harvested at the indicated time points and subjected to Western blot analysis as (a/b/c). (g) Effects of inhibitors on cell viability. H1299 cells were treated with 10 µM U0126, 8 µM sp600125 and 20 µM SB203580 for 24 h, respectively. The cell viability rate was determined by measuring the absorbance value at 450 nm after adding the CCK solution for 2 h.

To search for the upstream kinase(s) responsible for the induction of EGR1 in IBV-infected cells, ERK kinase1/2 inhibitor U0126, JNK inhibitor SP600125 and p38 inhibitor SB203580, were used to treat IBV-infected H1299 cells. As shown in Figure 4, treatment of IBV-infected H1299 cells with 10 µM U0126 almost completely blocked the induction of EGR1, whereas viral replication was only slightly affected (Figure 4(a)). It suggests that ERK1/2 may be responsible for upregulating EGR1 expression in IBV-infected cells as an upstream kinase. Treatment of IBV-infected H1299 cells with 8 µM of JNK inhibitor SP600125 and 20 µM of p38 inhibitor SB203580, respectively, showed significantly increased expression of EGR1 protein, even though the viral replication was slightly inhibited (Figure 4(b,c)). In addition, the effect of these inhibitors at the concentrations used in this study on the proliferation of H1299 cells was tested by measuring the absorbance value at 450 nm after adding the CCK solution for 2 h. As shown in Figure 4(g), no significant difference in the cell viability was observed in cells treated with inhibitors, compared to untreated cells (Mock).

To confirm that ERK1/2 is indeed an upstream kinase responsible for the induction of EGR1 in IBV-infected cells, ERK1/2-, JNK- and p38-knockdown H1299 cells were infected with IBV. The expression of EGR1 in ERK1/2-knockdown cells was drastically reduced, but the level of IBV N protein was not significantly affected at 20 and 24 hpi (Figure 4(d)). In ERK1/2-knockdown cells infected with PEDV and HCoV-229E, the expression of EGR1 was also significantly suppressed (Figure S2a,b). Taken together, these results confirm that ERK1/2 may function as an upstream kinase and/or positive regulator in the activation of EGR1 during coronavirus infection.

Furthermore, significantly more PARP cleavage was observed in ERK1/2-knockdown cells at 24 h post-IBV infection (Figure 4(d)), and slightly more PARP cleavage was also detected in ERK1/2-knockdown cells infected with PEDV and HCoV-229E, respectively (Figure S2a and S2b), reflecting different replication kinetics of these viruses in the knockdown cells.

Interestingly, more EGR1 expression was detected in JNK-knockdown cells infected with IBV at 16 hpi (Figure 4(e)), but no obvious difference was detected at other time points as well as in p38-knockdown cells infected with IBV (Figure 4(f)). In the knockdown cells infected with PEDV, significantly more EGR1 expression was detected in JNK-knockdown cells at all three time points (Figure S2c). Once again, no obvious difference in the EGR1 expression was observed in p38-knockdown cells infected with PEDV (Figure S2d). These results would support that JNK and/or p38 may function as negative regulators/competitors in the activation of EGR1 during coronavirus infection. This prompted the following studies on the involvement of the MAPKs-EGR1 pathway in regulating the expression of cytokines/chemokines in coronavirus-infected cells.

### Differential roles of the EGR1 induction in regulating host antiviral response in cells infected with different coronaviruses

**Figure 5.**
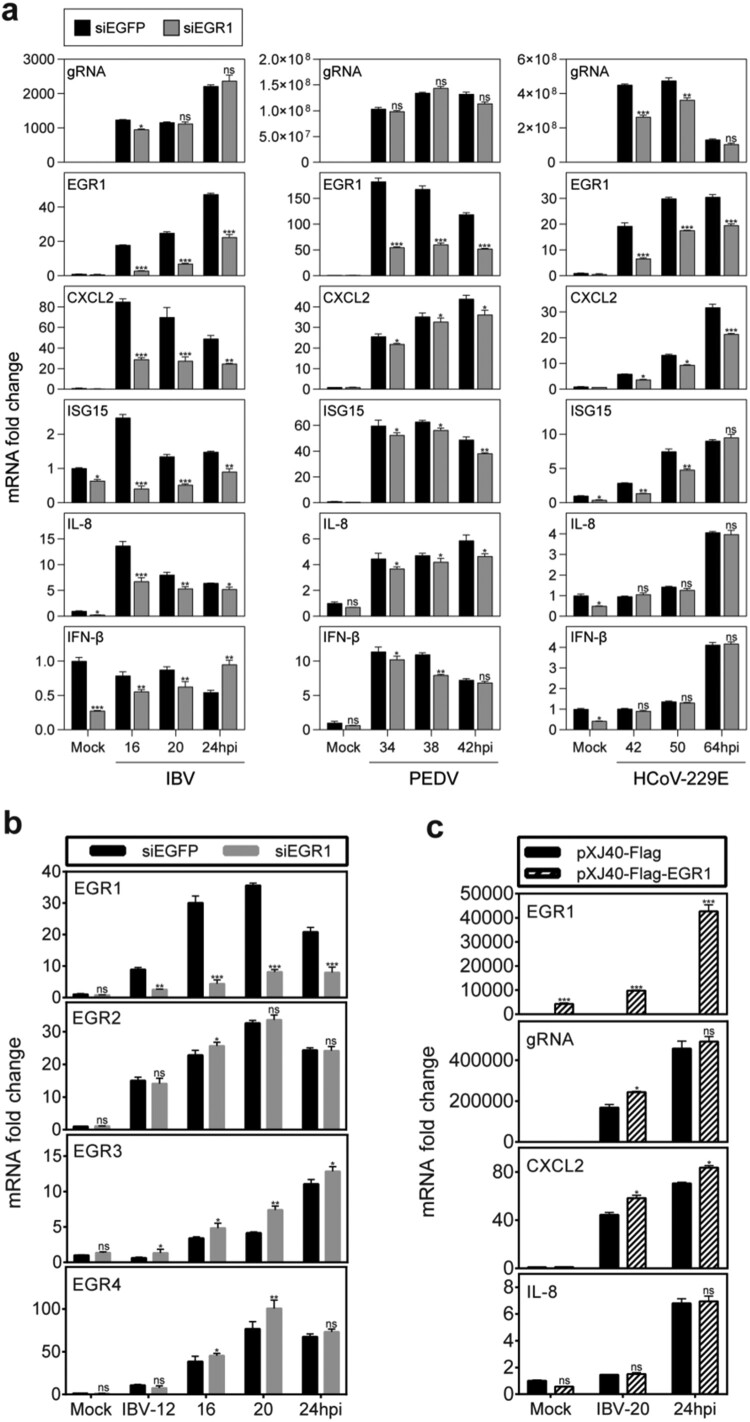
Regulation of the expression of cytokines and chemokines by coronavirus infection-induced EGR1 upregulation. (a) Differential effects of EGR1-knockdown on the expression of cytokines and chemokines in coronavirus-infected cells. H1299 cells were transfected with siEGFP and siEGR1, before infected with IBV/PEDV/229E at MOI∼2. Cell lysates were harvested at the indicated time points for RT-qPCR. The mRNA levels of EGR1, CXCL2, ISG15, IL-8 and IFN-β were determined by qPCR. Viral gRNA levels were determined as an indicator for IBV/PEDV/229E replication efficiency. (b) Effects of EGR1-knockdown on the expression of EGR2, EGR3 and EGR4 in IBV-infected cells. H1299 cells were treated as (a), Cells were harvested and analysed as (a). The mRNA levels of EGR1, EGR2, EGR3 and EGR4 were determined. (c) Effects of EGR1-overexpression on the expression of cytokines and chemokines in IBV-infected cells. H1299 cells were transfected with pXJ40-Flag and pXJ40-Flag-EGR1 before being infected with IBV or mock-infected (Mock). Cells were harvested and analysed as (a).

As a nuclear transcription factor, EGR1 regulates the expression of many cytokines in various physiological processes [30,31]. The regulatory roles of EGR1 in the induction of a number of antiviral cytokines/chemokines, including IFN-β, IL-8, ISG15 and CXCL2, were studied in EGR1-knockdown H1299 cells infected with IBV, PEDV and HCoV-229E, respectively. As shown in Figure 5(a), efficient knockdown of EGR1 was achieved, and similar levels of the IBV and PEDV genomic RNA were detected in the EGR1-knockdown cells. Induction of all the four cytokines/chemokines was significantly suppressed in the knockdown cells infected with both IBV and PEDV at most time points, but much more inhibitory effects were observed in the knockdown cells infected with IBV (Figure 5(a)). One exception was that the induction of IFN-β was increased in the knockdown cells infected with IBV at 24 hpi (Figure 5(a)). In cells infected with HCoV-229E, the viral genomic RNA was significantly reduced in EGR1-knockdown cells at 42 and 50 hpi (Figure 5(a)). Although the induction of the four cytokines/chemokines was also suppressed, it would be difficult to ascertain if this was due to the reduced viral replication.

The effect of EGR1 knockdown on the expression of other EGR family genes was investigated, showing varying levels of higher induction of EGR2, EGR3 and EGR4 transcripts in EGR1-knockdown cells infected with IBV (Figure 5(b)). Among them, much higher induction of EGR3 was observed in all time points in the knockdown cells, compared with the control (Figure 5(b)). The higher induction of these EGR family genes in EGR1-knockdown cells may functionally compensate for EGR1.

In cells overexpressing EGR1, the expression of CXCL2 was moderately increased, but the induction of IL-8 expression was not affected, probably due to the drastic upregulation of the endogenous EGR1 and other EGR family members in the infected cells (Figure 5(c)). Taken together, these results indicate that EGR1 may play a functional role in regulating the antiviral response against coronavirus infections.

## Discussion

**Figure 6.**
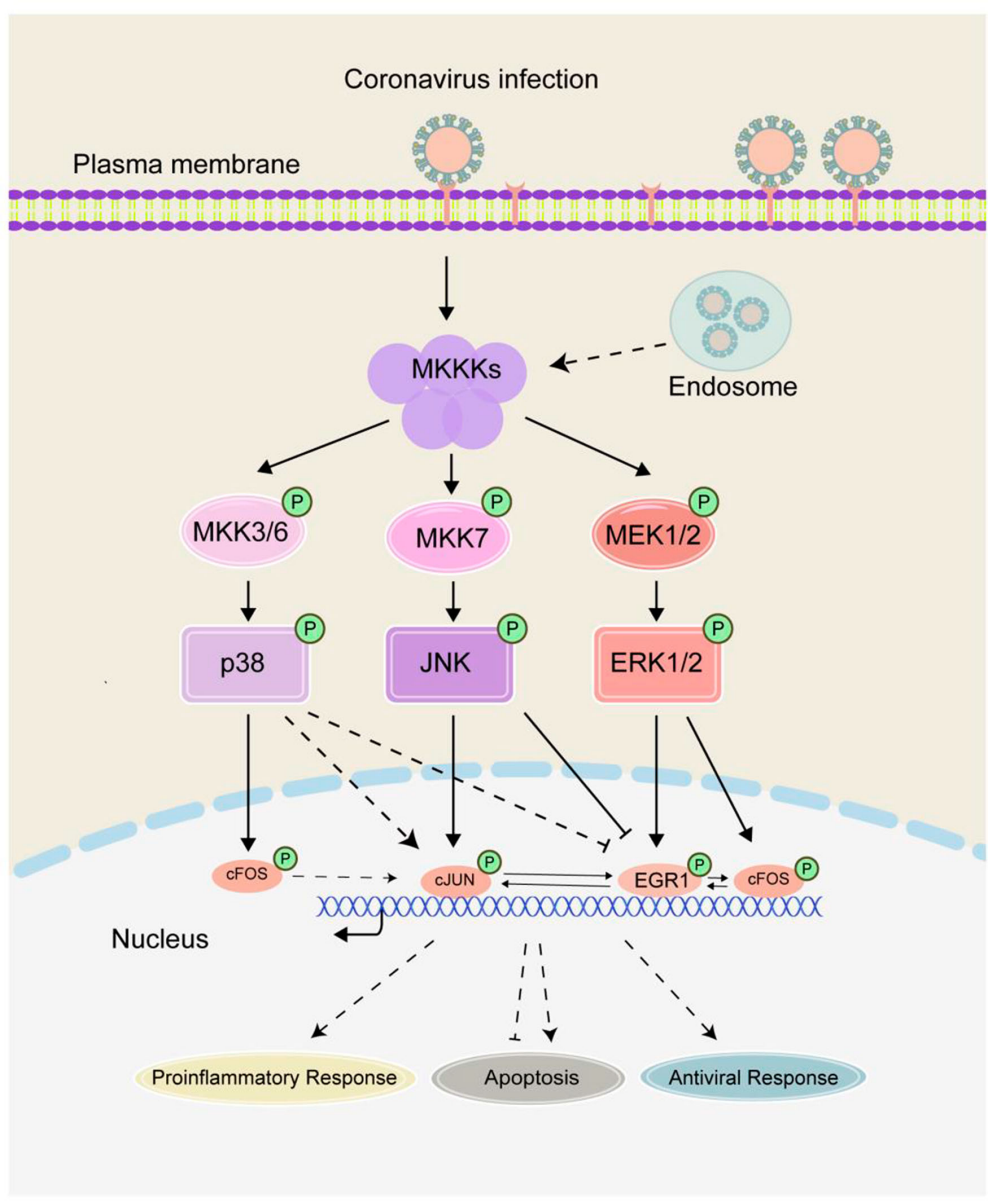
Current working model. The working model showing the activation of the MKKs-ERK1/2-EGR1 pathway and mutual regulation of ERG and AP-1 family genes during coronavirus infection. The functional impact of EGR1 activation on coronavirus-induced apoptosis and proinflammatory response is illustrated. Pointed and blunt arrows denote activation and suppression, respectively. “P” denotes phosphorylation. Dotted lines denote processes that are not fully characterized.

Virus invasion imposes a tremendous pressure on cell. In response to such external stimuli, the cell expresses a diversity of IEGs and other stress-related genes in order to modulate cellular pathways and biological processes. In our previous studies, we have shown that coronavirus infection induces the expression of AP-1 family genes cFOS and cJUN, modulating coronavirus infection-induced apoptosis and proinflammatory response [18–20]. In this study, another family of IEGs, the EGR family genes, especially EGR1, is confirmed to be significantly upregulated in cells infected with gammacoronavirus IBV, alphacoronaviruses PEDV and HCoV-229E, and betacoronavirus HCoV-OC43, revealing a common strategy shared among different genera of coronaviruses. This novel cellular regulatory network in early response to coronavirus infection is illustrated in Figure 6.

In fact, upregulation of EGR1 expression was found in cells infected with many different viruses. For example, Borna disease virus [32], human foamed virus [33], murine coronavirus mouse hepatitis virus [34], foot-and-mouth disease virus (FMDV) [35], and Venezuelan equine encephalitis virus [36]. It has been reported that EGR1 promotes the replication of vaccinia virus in starving fibroblasts by affecting the infectivity of virus particles [37]. On the other hand, overexpression of EGR1 was reported to inhibit the replication of FMDV in pig cells, and down-regulation of EGR1 significantly promoted the replication of FMDV [35]. The expression of EGR1 was significantly upregulated after Venezuelan equine encephalitis virus infection, and this upregulation was regulated by the ERK and PERK pathways, promoting cell death [36,38]. In this study, we showed that either overexpression or knockdown of EGR1 renders a minor effect on IBV replication in a time-course experiment up to 28 hpi. However, knockdown of EGR1 has a significant inhibitory effect on the replication of HCoV-229E in a time-course experiment up to 70 hpi. It is not clear if this is due to the much different replication courses of the two coronaviruses, but it appears that EGR1 may play either an enhancement or inhibitory role depending on the virus studied.

The MEK/ERK signalling pathway has been implicated to play a role in mediating EGR1 upregulation and activation [15,34]. This is consistent with our observation that a significant reduction in the upregulation of EGR1 was detected either in ERK1/2-knockdown cells or in the presence of an MEK/ERK inhibitor. On the contrary to the reported observation of JNK-mediated induction of EGR1 in stress response [39], this study showed that inhibition of either JNK or p38 enhanced the upregulation of EGR1 in coronavirus-infected cells, suggesting that JNK and p38 may function as negative regulators in coronavirus infection-induced upregulation of ERG1. The underlying mechanism is yet to be fully revealed, but it may point to the possibility that inhibition of JNK and p38 may reduce their competitive edge for the signals from their common upstream kinases with ERK1/2, leading to the enhanced upregulation of EGR1 in this context (Figure 6). Alternatively, JNK may regulate the expression of cJUN, which, in turn, regulates the expression of EGR1 (see below). If the former is the case, it would lend further support to the conclusion that ERK1/2 is the upstream kinase responsible for the activation of EGR1 in coronavirus-infected cells. Furthermore, as various regulatory mechanisms underlying the ERK1/2 activation/suppression were reported in IBV-infected cells in a number of studies [40,41], it would be interesting to further characterize the interaction between ERK1/2 and EGR1 during coronavirus infection. The two IEG family genes AP-1 and EGR are shown to be cross-activated and mutual regulated in coronavirus-infected cells in this study. This is consistent with a report by Hofmann that cJUN may function as a key factor in the transcriptional regulation of EGR1 [11], supporting that there is a link between stress-activated JNK-cJUN signalling pathway and EGR1 activation. However, it remains controversial whether EGR1 is the upstream activating molecule of JUN [42], or JUN is an upstream transcription factor of EGR1 [43]. The current study showed that in EGR1-knockdown cells infected with coronaviruses, the expression of cJUN was decreased, and in cJUN- and/or cFOS-knockdown and infected cells, the expression of EGR1 was also reduced. This points to a similar activation kinetics of EGR1 and cJUN/cFOS, and would indicate a simultaneous cross-activation of the two IEG family genes in the infected cells.

Through direct binding to the promoters of various apoptosis-inducing factors, such as BAX, NAG1 and PTEN, and stimulation of their expression, EGR1 was reported to induce apoptosis [44–46]. In pancreatic cancer cells, δ-tocoptrienol triggers the EGR1 expression through the JNK/cJUN pathway, and the upregulated EGR1 binds to the BAX promoter to activate BAX expression, leading to pancreatic cancer cell apoptosis [46]. EGR1 may modulate apoptosis through the unfolded protein response pathways in Venezuelan equine encephalitis virus-infected cells [36]. In this study, differential effects of EGR1-knockdown on coronavirus infection-induced apoptosis were observed in cells infected with different coronaviruses. In EGR1-knockdown cells infected with IBV and PEDV, apoptosis was significantly promoted, suggesting an anti-apoptotic function of EGR1. However, neither promotion nor inhibition of apoptosis was detected in H1299 cells infected with HCoV-229E. It appears that EGR1 exerts differential regulatory roles in cell apoptosis when the same cell types were infected with different coronaviruses.

EGR1 is reported to regulate inflammation and immune response [11,16]. The response of the immune system to stimulation is usually manifested by the high expression of inflammatory factors, and EGR1 expression is associated with the induction of several inflammatory mediators, including IL6, McP-1, CCL2 and TNFα, as well as some chemokines [17]. Lipopolysaccharide (LPS)-induced TNF-α expression is mediated by ERK1/2 activation of EGR1 in macrophage [16]. Baer et al. confirmed a connection between EGR1 and the interferon response genes (IRF1, IFIT1, IFIT2, ISG15, and ILF3) by RNA sequencing analysis [36]. In this study, the induction of IL-8, ISG15 and CXCL2 was significantly reduced in EGR1-knockdown cells infected with IBV, confirming the involvement of EGR1 in regulating the expression of these cytokines.

In summary, this study provides evidence on the cross-activation and mutual regulation of two IEG families during coronavirus infection. This would pave a way for further elucidation of events and the interplay between coronaviruses and their hosts at the immediate-early phase of the viral intrusion.

## Supplementary Material

Supplemental Material
