# Supplementary material for: Modulation of viral replication, apoptosis and antiviral response by induction and mutual regulation of EGR and AP-1 family genes during coronavirus infection
Source: Emerg Microbes Infect. 2022 Jul 4;11(1):1717–29. doi: 10.1080/22221751.2022.2093133 (PMC9262369; doi:10.1080/22221751.2022.2093133)
Supplement: Supplemental Material [file TEMI_A_2093133_SM6221.zip › Supplementary Figure Legends.docx]

## Figure S1. Upregulation of the EGR family genes in cells infected with PEDV, HCoV-229E and HCoV-OC43.

1. Upregulation of the EGR family genes at the mRNA level in cells infected with PEDV, HCoV-229E and HCoV-OC43. H1299 and/or Vero cells were infected with PEDV, HCoV-229E and HCoV-OC43 (MOI~2), respectively, and harvested at indicated time points for RT-qPCR. The genomic RNA levels of PEDV (PEDVgRNA), HCoV-229E (229EgRNA) and HCoV-OC43 (OC43gRNA), and the mRNA levels of EGR family genes (EGR1/EGR2/EGR3/EGR4) were determined by qPCR.
2. Upregulation of the EGR family genes at the protein level in cells infected with PEDV, HCoV-229E and HCoV-OC43. H1299 and Vero cells were infected with PEDV/HCoV-

229E/HCoV-OC43 (MOI~2), or UV-inactivated PEDV/HCoV-229E/HCoV-OC43. Cells were harvested at the indicated time points and subjected to Western blot analysis using indicated antibodies. Sizes of protein ladders in kDa were indicated on the left.

Figure S2. Identification of the upstream kinase(s) for EGR1 induction in PEDV/HCoV-229E-infected cells.

1. Suppression of EGR1 upregulation by knockdown of ERK1/2 in PEDV- or HCoV-229E- infected cells. H1299 cells were transfected with siEGFP and siERK1/2, before infected with PEDV/HCoV-229E. Cells were harvested at the indicated time points. Western blot analysis was performed using indicated antibodies. Sizes of protein ladders in kDa were indicated on the left.
2. Effects of JNK- or p38-knockdown on the expression of EGR1 in PEDV-infected cells. H1299 cells were transfected with siEGFP and siJNK/p38, before infected with PEDV. Cells were harvested at the indicated time points and subjected to Western blot analysis as (a/b).
