# Supplementary figures and images for: Modulation of viral replication, apoptosis and antiviral response by induction and mutual regulation of EGR and AP-1 family genes during coronavirus infection
Source: Emerg Microbes Infect. 2022 Jul 4;11(1):1717–29. doi: 10.1080/22221751.2022.2093133 (PMC9262369; doi:10.1080/22221751.2022.2093133)

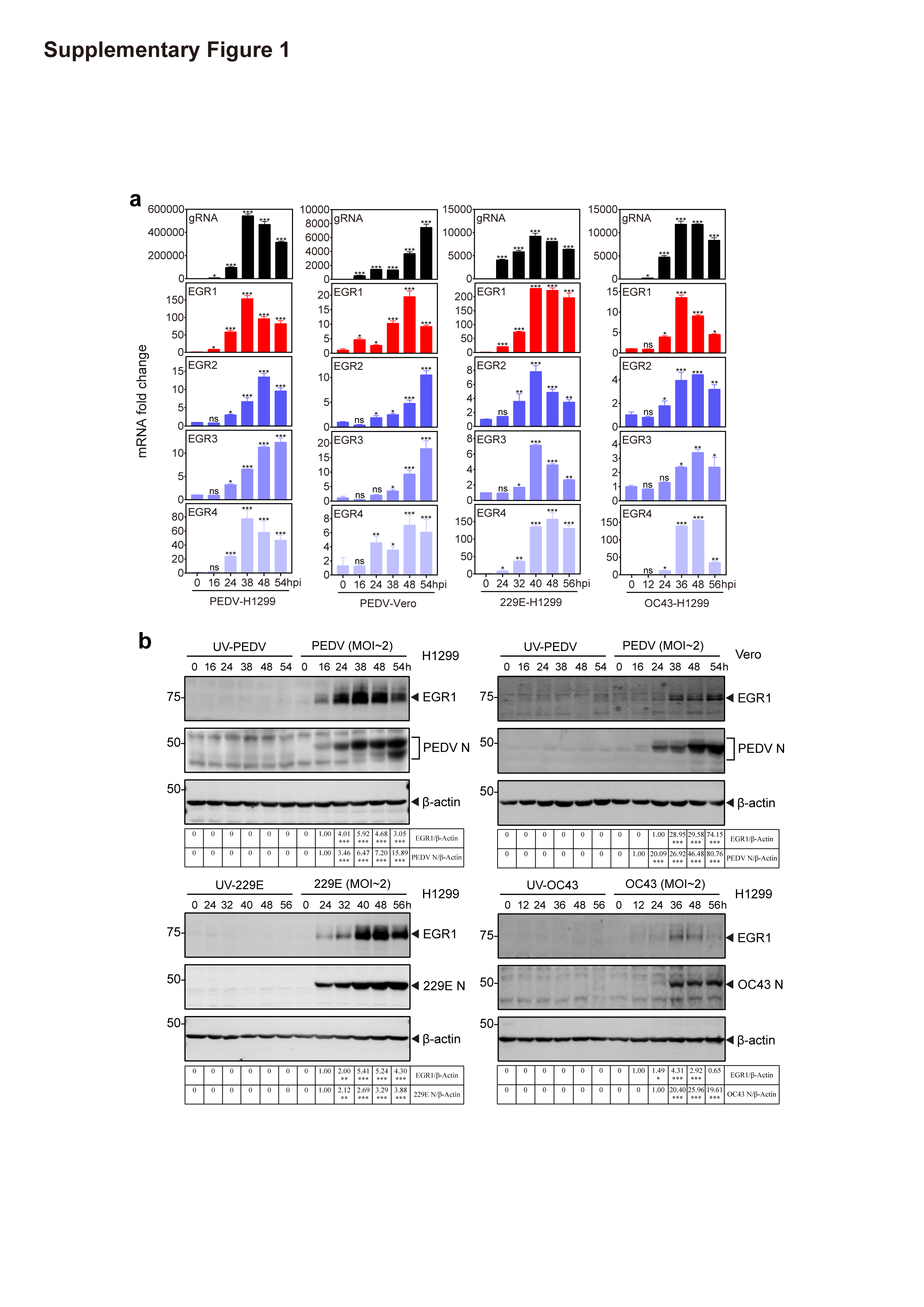

Supplement: Supplemental Material [file TEMI_A_2093133_SM6221.zip › Figure_S1.tiff]

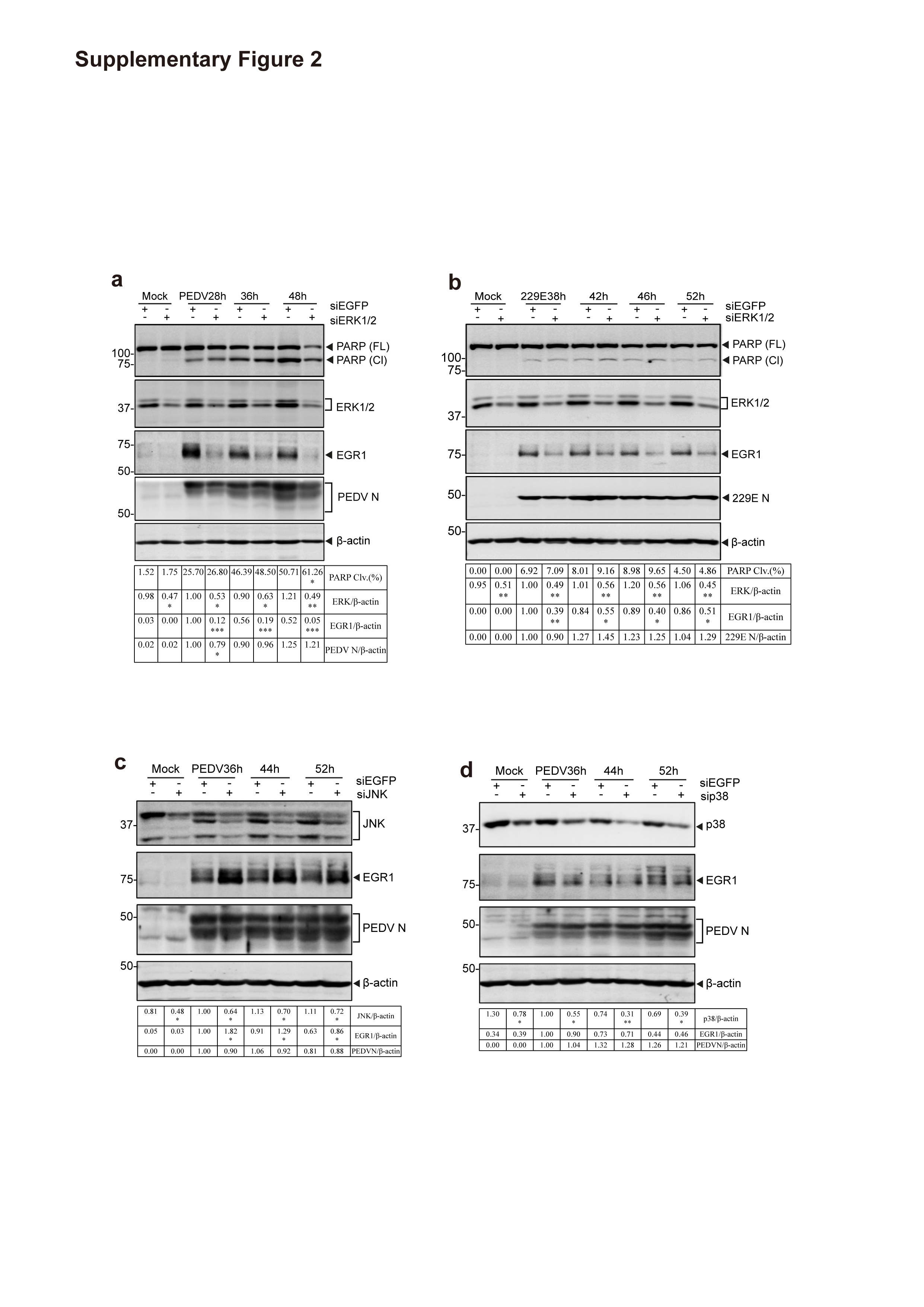

Supplement: Supplemental Material [file TEMI_A_2093133_SM6221.zip › Figure_S2.tiff]
